# *Spodoptera exigua* Multiple Nucleopolyhedrovirus Increases the Susceptibility to Insecticides: A Promising Efficient Way for Pest Resistance Management

**DOI:** 10.3390/biology12020260

**Published:** 2023-02-06

**Authors:** Shuxing Zhou, Jinming Zhang, Ya Lin, Xiaowei Li, Min Liu, Muhammad Hafeez, Jun Huang, Zhijun Zhang, Limin Chen, Xiaoyun Ren, Wanying Dong, Yaobin Lu

**Affiliations:** 1 State Key Laboratory for Managing Biotic and Chemical Threats to the Quality and Safety of Agro-Products, Institute of Plant Protection and Microbiology, Zhejiang Academy of Agricultural Sciences, Hangzhou 310021, China; 2 College of Chemistry and Life Sciences, Zhejiang Normal University, Jinhua 321004, China; 3 State Key Laboratory of Rice Biology & Ministry of Agriculture Key Laboratory of Agricultural Entomology, Institute of Insect Sciences, Zhejiang University, Hangzhou 310058, China; 4 Institute of Bio-Interaction, Xianghu Laboratory, Hangzhou 311258, China

**Keywords:** *Spodoptera exigua*, *S. exigua* multiple nucleopolyhedrovirus, insecticides, combined application, pest resistance management

## Abstract

**Simple Summary:**

The beet armyworm *Spodoptera exigua* (Hübner) is a polyphagous pest of numerous cultivated crops including potatoes, beans, asparagus, cotton, etc., causing considerable economic losses in crop production worldwide. Currently, the use of chemical insecticides is the most commonly used method to control this pest, however, the overuse of them results in insecticide resistance, environmental pollution and toxicity to other non-target organisms. Our results indicated that *S. exigua* field populations had developed resistance to almost half of the eleven commonly used insecticides with different modes of action. Thus, it is extremely urgent to seek an efficient strategy for insecticide resistance management. We found that the combined application of the specific entomopathogen of *S. exigua* (*Spodoptera exigua* multiple nucleopolyhedrovirus, SeMNPV) reduced insecticide resistance or even recovered the susceptibility to insecticides and enhanced the toxicity obviously in both laboratory experiments and field trials. These findings are valuable to provide a promising efficient way for improving insecticide resistance management strategy and an environmentally friendly approach for pest management with the combined application of nucleopolyhedroviruses and insecticides.

**Abstract:**

*Spodoptera exigua* is a polyphagous pest of diverse crops and causes considerable economic losses. The overuse of chemical insecticides for controlling this pest results in insecticide resistance, environmental pollution and toxicity to other non-target organisms. Therefore, a sustainable and efficient way for pest management is urgently required. In this study, laboratory bioassays of eleven commonly used insecticides, the specific entomopathogen of *S. exigua* (*Spodoptera exigua* multiple nucleopolyhedrovirus, SeMNPV), and SeMNPV-insecticide combinations against the *S. exigua* laboratory population and two field populations were tested. Our results indicated that the two field populations had developed resistance to almost half of the tested insecticides, while SeMNPV had good virulence in all populations. Interestingly, the combined use of SeMNPV enhanced the toxicity of the tested insecticides against all populations to a different extent and considerably reduced the insecticide resistance of *S. exigua* field populations or even recovered the susceptibility to above insecticides. Furthermore, the field trial showed that the combined application of SeMNPV contributed to promoting the control efficacy of emamectin benzonate and chlorfenapyr. These results provide a promising efficient way for pest resistance management and an environmentally friendly approach for controlling *S. exigua* with the combined application of nucleopolyhedroviruses and insecticides.

## 1. Introduction

The beet armyworm *Spodoptera exigua* (Hübner) (Lepidoptera: Noctuidae) is a polyphagous pest of numerous cultivated crops including potatoes, tomatoes, beans, peas, asparagus, tobacco, cotton, cereals, etc. [1,2,3]. Due to its worldwide distribution and polyphagous behavior, this pest causes considerable economic losses in crop production worldwide [2,3]. Currently, the use of chemical insecticides is the most commonly used method to control this pest [3]. However, the extensive use of synthetic insecticides not only exhibits harmful effects on the environment, non-target organisms and human health but also leads to the development of insecticide resistance [4,5,6]. Once insect pests have developed a high level of resistance to different groups of insecticides, their efficacy is directly impaired [6,7,8,9,10]. It has been reported that *S. exigua* exhibited significant levels of resistance to many chemical insecticides, for example, chlorantraniliprole, emamectin benzoate, spinosad, tebufenozide, cypermethrin, chlorfluazuron, indoxacarb, chlorpyrifos, chlorfenapyr, methoxyfenozide, tetraniliprole, beta-cypermethrin, lambda cyhalothrin, metaflumizone, abamectin and cyantraniliprole in China [11,12,13,14,15].

To resolve chemical insecticide resistance problems, an increasing number of studies have been conducted to develop and commercialize microbial insecticides [16,17,18]. Among them, entomopathogen nucleopolyhedroviruses (NPVs) have been reported to be effective alternatives to chemical insecticides against lepidopteran pests [17,19,20,21,22].

NPV (family *Baculoviridae*, genus *Alphabaculovirus*) has been developed as an effective biological pesticide to control lepidopteran pests in agriculture, horticulture and forestry in light of the advantages of host specificity, harmlessness to the environment and non-target organisms [17,23,24,25]. Nevertheless, the slow speed of kill limits its extensive application [22,25]. Considering the fast acting of chemical insecticides, combined application of NPVs with low concentrations of chemical insecticides might be considered as an efficient approach to achieve the goal of excellent effect on killing pests, reduction in environmental pollution and protection of non-target organisms [22]. Previous studies have supported this idea: *Autographa californica* multiple nucleopolyhedrovirus (AcMNPV) showed a synergistic effect with emamectin or metaflumizone; the synergistic effect was also shown between *S. litura* nucleopolyhedrovirus (SpltNPV) and chlorantraniliprole in *S. exigua* [26,27]. Similarly, *S. littoralis* nucleopolyhedrovirus (SpliNPV) exhibited synergy with azadirachtin, emamectin and spinosad in *S. littoralis* [26,28]. SpltNPV in combination with emamectin benzoate, chlorantraniliprole, flubendiamide and spinosad exhibited synergistic interaction against *S. litura* [27,29,30,31]. Further research indicated that *S. frugiperda* multiple nucleopolyhedrovirus (SfMNPV) had synergy with spinosad against *S. frugiperda* [32]; *Helicoverpa armigera* nucleopolyhedrovirus (HaNPV) had synergistic effects with spinetoram or emamectin benzoate against *H. armigera* [33]; *Bombyx mori* nucleopolyhedrovirus (BmNPV) showed a synergistic interaction with phoxim in *B. mori* [34].

Consequently, the combined application of *S. exigua* multiple nucleopolyhedrovirus (SeMNPV) with insecticides might work effectively for preventing the overuse of insecticides and improving insecticide resistance management strategy. To test our hypothesis, we investigated the effect of SeMNPV, eleven commonly used chemical insecticides with different modes of action and their combined application against *S. exigua* in both laboratory experiments and field trials. Our study will provide a promising efficient way for insecticide resistance management and an environmentally friendly approach for pest management.

## 2. Materials and Methods

### 2.1. Insects

The *Spodoptera exigua* laboratory population (Lab) was provided by Henan Jiyuan Baiyun Industry Co., Ltd. (Jiyuan, China) in 2017; the population was reared on an artificial diet in a climate room (26 ± 1 °C, 50% ± 10% relative humidity and a photoperiod of 14L:10D), without exposure to any insecticides for more than 30 generations before the start of the experiments. The two field populations referred to as PH and TX populations were collected from asparagus fields of Pinghu and Tongxiang, Zhejiang Province, China during the summer of 2020, respectively, and subsequently reared under the condition mentioned above until pupation. The pupae (1 d before adult emergence) were transferred into a cylindrical container containing white filter paper for egg collection. The emerged adults were supplied with the 10% honey solution as a food source. The third instar larvae of F1 generation from the two field populations were used for subsequent experiments.

### 2.2. Determination of LC_50_ of Insecticides

The leaf-dip method was applied for the determination of LC_50_ of insecticides [35]. Eleven insecticides with different mode of action were selected from commonly used insecticides against *S. exigua* in China (Table 1). All tested insecticides were firstly dissolved by acetone to acquire 1000 mg/L stock solution, then it was serially diluted using distilled water containing 0.1% Triton X-100 to obtain insecticide dilutions with six to seven concentration gradients (treatments) for toxicity bioassays. The ones treated with distilled water containing 0.1% Triton X-100 were considered as control. Fresh leaf discs of cabbage (4 cm in diameter) were cut and dipped in each serial dilution of tested insecticide for 10 s and air dried at 25 °C for 1 h. Leaf discs after drying were placed in 6.5 cm-diameter plastic Petri dishes along with moist filter paper to prevent desiccation before insect exposure, respectively. Five larvae were transferred to the leaf disc in a Petri dish as one replicate, and ten replicates were performed for each concentration in each tested insecticide. The Petri dishes were covered and transferred into a climate chamber at 26 ± 1 °C, 50% ± 10% relative humidity and a photoperiod of 14:10 (L:D). According to the different action modes of insecticides, larval mortality was recorded at 96 h after exposure to four insect growth regulators, and 48 h after exposure to the rest seven insecticides (chlorfenapyr, indoxacarb, chlorantraniliprole, cyantraniliprole, spinosad, spinetoram and emamectin benzonate). Larval mortality was recorded with concern to those which were unable to move from a gentle stimulus with a fine brush.

### 2.3. Determination of LC_25_ and LC_50_ of SeMNPV

The lethal and sub lethal concentrations (LC_25_ and LC_50_) of SeMNPV were determined by the method described by Allahyari et al. [36]. SeMNPV with the concentration of 3 × 10^10^ OBs/mL was supplied by Henan Jiyuan Baiyun Industry Co., Ltd. Firstly, SeMNPV was serially diluted in distilled water to acquire dilutions with six to seven concentration gradients (treatments) for bioassays. Following the method mentioned in Section 2.2, the mortality of third instar larvae was recorded after 48 h, 72 h, 96 h and 120 h after exposure to SeMNPV. Leaf discs treated with distilled water were considered as control. Ten replications were used for each concentration of SeMNPV.

### 2.4. Toxicity of Insecticides Combining with SeMNPV

SeMNPV was diluted in distilled water containing 0.1% Triton X-100 to obtain the solution containing LC_25_ concentration of SeMNPV. Subsequently, the solution containing SeMNPV was used for diluting the eleven insecticides with five to seven concentration gradients for bioassays. Determination of LC_50_ of insecticide combined with SeMNPV followed the method described in Section 2.2. Ten replicates were performed for each concentration in each tested insecticide. The ratio of enhanced toxicity was calculated by dividing LC_50_ of insecticides without SeMNPV by LC_50_ of insecticides with SeMNPV [37].

### 2.5. Field Trial

In the laboratory experiment, the toxicity was enhanced most obviously between SeMNPV and emamectin benzonate or chlorfenapyr against *S. exigua* in the PH population. Consequently, these two insecticides were selected for the field trial to confirm the feasibility of reduction in insecticide use with the combination of SeMNPV in asparagus field in Pinghu. Chlorfenapyr (10% SC, Shandong Weifang Pesticide Co., Ltd. (Weifang, China)) and emamectin benzonate (5% WG, Huizhou Yinnong Technology Co., Ltd. (Huizhou, China)) were applied in field trial. Additionally, during the field trial, the weather was either cloudy or sunny (16 °C–27 °C). Six treatments were designed to test whether SeMNPV could enhance the efficacy of chlorfenapyr and emamectin benzonate against *S. exigua* (Table 2). The experiments were conducted using a randomized complete block design with four replications. The number of survival *S. exigua* larvae on 10 asparagus per block was investigated after 3 d, 6 d or 10 d exposure after treatments. Additionally, the field efficacy was evaluated by the mortality of larvae.

### 2.6. Data Analysis

The data of larval mortality in Section 2.2, Section 2.3 and Section 2.4 were subjected to probit analysis using PoloPlus software, version 1.0, LeOra Software Company (Berkeley, CA, USA) to calculate the LC_50_ and LC_25_ values [35]. The resistance ratio (RR) was determined by dividing the LC_50_ of the field population by the LC_50_ of the Lab population. Based on resistance ratios, resistance levels were classified into five levels including susceptibility (RR < 5), low resistance (5 ≦ RR < 10), moderate resistance (10 ≦ RR < 40), high resistance (40 ≦ RR < 160) and extremely high resistance (RR ≧ 160) [12]. The field efficacy was analyzed by the generalized linear model (GLM) using SPSS statistics software, version 18.0, IBM Corporation (Armonk, NY, USA).

## 3. Results

### 3.1. Determination of LC_50_ of Insecticides

To investigate the current status of the resistance of *S. exigua* to eleven commonly used insecticides in asparagus fields, the toxicity of these insecticides to two field populations (PH and TX) was determined. As shown in Table 3, compared to the Lab population, the two field populations developed different levels of resistance to almost half of the tested insecticides. To be specific, the PH population exhibited high resistance to emamectin benzonate (44.57-fold), moderate resistance to spinetoram (15.37-fold) and indoxacarb (11.38-fold), low resistance to chlorfenapyr (7.82-fold) and chlorfluazuron (6.06-fold), respectively. Similarly, the TX population displayed moderate resistance to chlorantraniliprole (22.61-fold), indoxacarb (15.94-fold) and emamectin benzonate (10.57-fold), as well as low resistance to chlorfenapyr (6.62-fold), spinetoram (6.58-fold) and chlorfluazuron (5.49-fold). Therefore, it is extremely urgent to seek strategies for reducing the resistance of *S. exigua* to these insecticides.

### 3.2. Determination of LC_25_ and LC_50_ of SeMNPV

In view of previous research about the synergy between nucleopolyhedrovirus and insecticides against pests [26,27,29,33,34], the specific entomopathogen of *S. exigua*, SeMNPV was selected for the further combined application. SeMNPV had good virulence against three *S. exigua* populations, with the highest and lowest virulence in Lab and TX populations, respectively (Table 4). Moreover, LC_25_ and LC_50_ values decreased with the increase in infection time of SeMNPV and the lab population responded faster to SeMNPV (Table 4).

### 3.3. Toxicity of Insecticides Combining with SeMNPV against S. exigua

**Figure 1:**
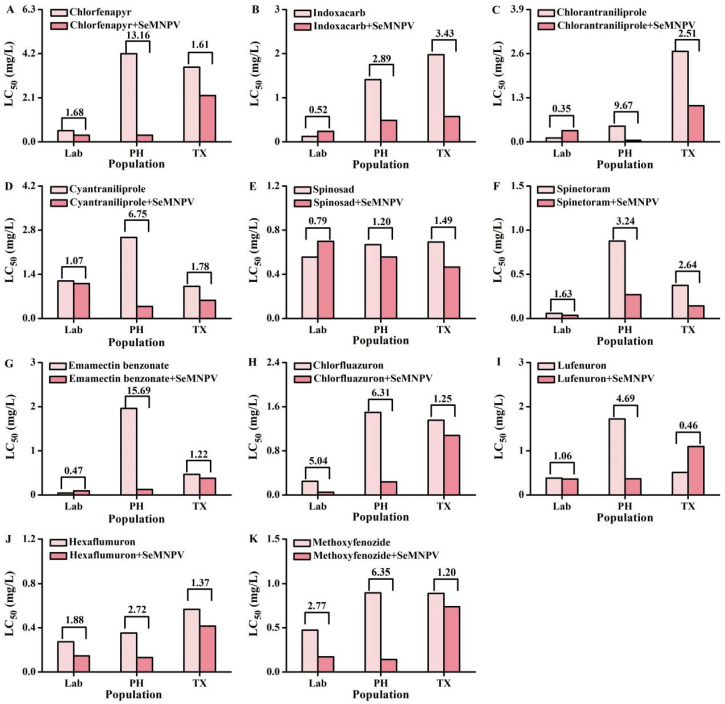
SeMNPV enhanced the toxicity of insecticides against *S. exigua*. LC_50_ of chlorfenapyr (**A**), indoxacarb (**B**), chlorantraniliprole (**C**), cyantraniliprole (**D**), spinosad (**E**), spinetoram (**F**), emamectin benzonate (**G**), chlorfluazuron (**H**), lufenuron (**I**), hexaflumuron (**J**), methoxyfenozide (**K**) against three *S. exigua* population (Lab, PH and TX) with or without SeMNPV infection. The numbers in the above bars represent the ratio of enhanced toxicity.

Results showed that the LC_50_ of insecticides decreased to a different extent among different *S. exigua* populations (Figure 1). Specifically, for the Lab population, the toxicity of seven insecticides (chlorfluazuron, methoxyfenozide, hexaflumuron, chlorfenapyr, spinetoram, cyantraniliprole and lufenuron) was enhanced by SeMNPV infection. The highest ratio of enhanced toxicity was observed in chlorfluazuron, where the efficacy exhibited 5.04-fold in comparison to a single insecticide application (Figure 1H). For the TX population, except for lufenuron, the toxicity of the rest ten insecticides was increased by combined use of SeMNPV, with indoxacarb showing the greatest enhancement by SeMNPV at 3.43-fold (Figure 1B). For the PH population, SeMNPV enhanced the toxicity of all the eleven insecticides, with the efficacy elevated over 10 times for emamectin benzonate and chlorfenapyr, exhibiting 15.69- and 13.16-fold higher compared with single insecticide, respectively (Figure 1A,G). Increased toxicity of insecticides was observed more obviously in the two field populations compared to the Lab population. Interestingly, the insecticide resistance of two field populations was dramatically decreased by SeMNPV (Table 5). After exposure to SeMNPV, the PH population exhibited susceptibility to all the tested insecticides and the TX population showed susceptibility to nine of them, except for chlorantraniliporle and emamectin benzonate, whose resistance ratio was decreased from 22.61 to 9.03 and from 10.57 to 8.61, respectively (Table 5). Therefore, SeMNPV might be a promising efficient way for the insecticide resistance management of *S. exigua*, thus resulting in a reduction in insecticide use.

### 3.4. Field Trial

**Figure 2:**
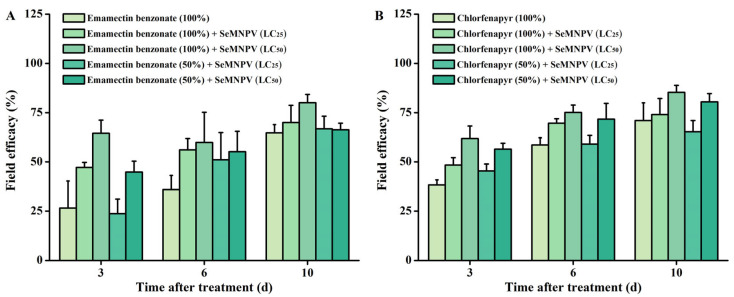
SeMNPV enhanced the field efficacy of emamectin benzonate and chlorfenapyr against *S. exigua*. The control efficacy of emamectin benzonate (**A**) and chlorfenapyr (**B**) with or without SeMNPV against *S. exigua*. Emamectin benzonate (100%) and emamectin benzonate (50%) represented 100% and 50% recommended dosage of emamectin benzonate. Chlorfenapyr (100%) and chlorfenapyr (50%) represented 100% and 50% recommended dosage of chlorfenapyr.

Among the eleven insecticides, the greatest reduction in LC_50_ occurred in emamectin benzonate and chlorfenapyr in the PH population when combined with SeMNPV (Figure 1); therefore, these two insecticides were selected for field trial. After 3 days post-treatment (dpt), the treatment significantly affected the field efficacy of emamectin benzonate (Wald = 14.082, *p* = 0.007) and chlorfenapyr (Wald = 12.522, *p* = 0.014). The field efficacy of emamectin benzonate and chlorfenapyr was 26.61 ± 13.76% and 38.33 ± 2.60%, respectively (Figure 2). Surprisingly, combined application of SeMNPV (LC_25_ and LC_50_) with emamectin benzonate exhibited 1.77- and 2.43-fold higher field efficacy of single emamectin benzonate, moreover, significant difference was found between emamectin benzonate and combination of LC_50_ SeMNPV with emamectin benzonate (Wald = 8.731, *p* = 0.003) (Figure 2A). Similarly, the field efficacy of the combined use of chlorfenapyr and SeMNPV (LC_25_ or LC_50_) was enhanced 1.26- and 1.62-fold as compared with the application of sole chlorfenapyr; furthermore, there was a significant difference between chlorfenapyr and combination of LC_50_ SeMNPV with chlorfenapyr (Wald = 10.416, *p* = 0.001) (Figure 2B). Moreover, it is interesting that even if the reduction in the use of these two insecticides reached up to 50%, the field efficacy was unaffected or even improved in the condition of combined use of SeMNPV. At 6 dpt and 10 dpt, the same trend was observed; however, there was no significant difference between the treatment and field efficacy of emamectin benzonate (Wald = 4.931, *p* = 0.294; Wald = 5.732, *p* = 0.220) and chlorfenapyr (Wald = 8.245, *p* = 0.083; Wald = 5.434, *p* = 0.246) (Figure 2). In conclusion, the combined application of SeMNPV enhanced the field efficacy of emamectin benzonate and chlorfenapyr against *S. exigua,* thus providing a promising way for reducing the use of these two chemical insecticides.

## 4. Discussion

In this study, we found that *S. exigua* field populations have developed resistance to almost half of eleven insecticides with different modes of action, but SeMNPV still had good virulence against these populations. Additionally, SeMNPV application in combination with chemical insecticides reduced insecticide resistance against *S. exigua* and increased the efficacy of the insecticides.

Numerous studies have reported that the resistance of this pest to chlorantraniliprole, indoxacarb, spinosad, chlorfenapyr, abamectin, emamectin benzoate, methoxyfenozide, chlorfluazuron, chlorpyrifos, beta-cypermethrin, hexaflumuron and cyantraniliprole presented a rising trend in the field [12,15,38,39,40]. Likewise, our results demonstrated that the PH and TX field populations of *S. exigua* have developed significant resistances to emamectin benzonate, chlorantraniliprole, spinetoram, indoxacarb, chlorfenapyr and chlorfluazuron (Table 3), due to the indiscriminate of these insecticides in the field.

According to previous studies, the application of NPVs in combination with insecticides revealed a synergistic effect against many pests [26,27,29,33,34], which enlightened us on the reduction in insecticide resistance by SeMNPV in *S. exigua*. Other investigations have found that when *S. exigua* is infected with SeMNPV, occlusion bodies (OBs) degrade in an alkaline environment and release occlusion-derived virus (ODV) virions to infect midgut cells, followed by the formation of budded virions (BVs) and the OBs of further cells and make *S. exigua* larvae liquefy [23,41,42]. It is a complicated process of SeMNPV infection, which takes a long time. Therefore, the virulence of SeMNPV increased with increasing infection time. The lab population reared without exposure to any insecticides or SeMNPV in laboratory conditions was more sensitive to SeMNPV compared to the two field populations with long-term exposure to various insecticides (including SeMNPV) in asparagus fields, which leads to faster responses to SeMNPV in the lab population. In addition, temperature, food and other environmental factors are different from the lab condition and field condition, which may lead to some physiological differences related with the resistance to SeMNPV and insecticides between the laboratory population and field population; thereby, increased susceptibility to SeMNPV and insecticides was observed in laboratory population. Our results indicated that SeMNPV had good virulence to all three *S. exigua* populations, however, the intensity of virulence was different among the three populations (Table 4). The difference among different populations was supported by earlier reports: both median lethal dose and time-mortality curves were different between two *S. exigua* colonies, which may be due to the different genetic backgrounds of these populations [43,44]. Because of SeMNPV’s high virulence against *S. exigua*, it was used in our study to reduce insecticide resistance and consumption.

Furthermore, our results demonstrated that the combined application of SeMNPV increased the susceptibility to all the eleven insecticides in the PH population, ten of them in the TX population and seven of them in the Lab population (Figure 1). Moreover, the insecticide resistance of the two field populations was considerably decreased after SeMNPV infection (Table 5). The synergistic effect between other lepidopteran NPVs and numerous insecticides was uncovered, for example, SpltNPV and chlorantraniliprole in *S. exigua* [27], SpliNPV and spinosad in *S. littoralis* [28], SfMNPV and spinosad in *S. frugiperda* [32], SpltNPV and emamectin benzoate, chlorantraniliprole or spinosad in *S. litura* [27,29,31], HaNPV and spinetoram or emamectin benzoate in *H. armigera* [33], AcMNPV and emamectin or metaflumizone in *S. exigua* [26], SpliNPV and azadirachtin or emamectin in *S. littoralis* [26], SfMNPV and azadirachtin in *S. frugiperda* [45], SpltNPV and flubendiamide or azadirachtin in *S. litura* [30,46], and BmNPV and phoxim in *B. mori* [34]. The synergy may be responsible for our results, however, it needs to be further verified in the future. In previous research, it has been reported that SeMNPV infection enhanced the permeability of peritrophic matrix (PM) by changing the expression of PM-related genes such as up-regulated expression of chitin deacetylases, and suppressed the immune system by the down-regulated expression of detoxification and certain antiviral-related genes in the midgut of *S. exigua* larvae [47,48]. It is well known that cuticle and detoxification enzymes in the midgut play a pivotal role in the development of insecticide resistance [49,50,51,52,53]. However, the biological explanation for these interactions is unknown and the mechanism should be explored in the future. In contrast, the antagonistic effect was observed between NPVs and insecticides in some cases, for example, HaNPV with a certain dose of spinosad, spinetoram or emamectin benzoate in *H. armigera* [33,54], and SfMNPV and spinosad with a certain concentration in *S. frugiperda* [32]. Therefore, concentration could be considered as an important factor in the interaction effect between NPVs and insecticides, which may account for no obvious enhanced toxicity of some insecticides combining with SeMNPV in the Lab population and TX population in this study. Meanwhile, it is interesting that the ratio of enhanced toxicity varied among the three populations in our research (Figure 1). A previous study carried out by Ahmad et al. supported this result, who suggested that SpltNPV exhibited different interaction effects between spinosad on the larval mortality of three different geographical populations of *S. litura* [31]. The different degrees of enhanced toxicity between SeMNPV and the same insecticide in three populations may be due to their different sensitivity to SeMNPV.

Our field trial showed that the combined application of SeMNPV enhanced the field efficacy of emamectin benzonate and chlorfenapyr against *S. exigua* (Figure 2), which was in line with the above laboratory experiment (Figure 1). Likewise, previous studies are in agreement with our results, suggesting the enhanced field efficacy of NPV-insecticide mixtures against lepidopteran pests in the field, for example, SpltNPV and spinosad against *S. litura* in cotton [55]; SpltNPV and flubendiamide or *Bacillus thuringiensis* against *S. litura* on cauliflower [56]; HaNPV and spintoram or emamectin benzoate against *H. armigera* in cotton field [57]; SeMNPV and *B. thuringiensis* against *S. exigua* on tomato [58]. Even if the reduction in the use of insecticides reached up to 50%, the field efficacy was unaffected when combining the use of SeMNPV (Figure 2), which provides a promising way for reducing the use of insecticides. An SeMNPV infection-induced increase in the susceptibility of the *S. exigua* field population may be responsible for this result. Confirming the field efficacy of NPV-insecticide mixtures is essential in improving the insecticide resistance management strategy; therefore, more field trials should be carried out.

## 5. Conclusions

Our results demonstrated that the combined application of SeMNPV considerably reduced insecticide resistance or even recovered the susceptibility to insecticides and improved the efficacy of insecticide against *S. exigua* in both laboratory experiments and field trials. Hence, the combined use of NPVs and insecticides provides a promising efficient way for pest resistance management and a more environmentally friendly approach for controlling pests with less consumption of chemical pesticides in the field.

## Figures and Tables

**Table 1:** Insecticides used for experiments.

Type	Common Name	Active Ingredient Content	Supplier
Pyrroles	Chlorfenapyr	98%	Hangzhou Lancheng Technology Co., Ltd. (Hangzhou, China)
Oxadiazine	Indoxacarb	97%	
Amides	Chlorantraniliprole	96%	
	Cyantraniliprole	94%	
Macrolides	Spinosad	90%	
	Spinetoram	94%	
	Emamectin benzonate	70%	
Insect growth regulators	Chlorfluazuron	96%	
	Lufenuron	97%	
	Hexaflumuron	97.6%	
	Methoxyfenozide	98%	

**Table 2:** Treatments designed for the field trial.

Insecticide	Treatment
Chlorfenapyr and SeMNPV	Water (control)
	Chlorfenapyr (1800 mL/hm^2^, recommended dose)
	SeMNPV (8.58 × 10^6^ OBs/mL) + chlorfenapyr (900 mL/hm^2^)
	SeMNPV (1.91 × 10^7^ OBs/mL) + chlorfenapyr (900 mL/hm^2^)
	SeMNPV (8.58 × 10^6^ OBs/mL) + chlorfenapyr (1800 mL/hm^2^)
	SeMNPV (1.91 × 10^7^ OBs/mL) + chlorfenapyr (1800 mL/hm^2^)
Emamectin benzonate and SeMNPV	Water (control)
	Emamectin benzonate (900 g/hm^2^, recommended dose)
	SeMNPV (8.58 × 10^6^ OBs/mL) + emamectin benzonate (450 g/hm^2^)
	SeMNPV (1.91 × 10^7^ OBs/mL) + emamectin benzonate (450 g/hm^2^)
	SeMNPV (8.58 × 10^6^ OBs/mL) + emamectin benzonate (900 g/hm^2^)
	SeMNPV (1.91 × 10^7^ OBs/mL) + emamectin benzonate (900 g/hm^2^)

**Table 3:** Resistance of two field populations of *Spodoptera exigua* against eleven insecticides.

Insecticide	Population	LC_50_ (mg/L) (95% CI)	Slope ± SE	χ^2^ (df)	RR
Chlorfenapyr	Lab	0.537 (0.389–0.728) b	1.139 ± 0.137	1.445 (5)	—
	PH	4.198 (3.422–5.189) a	1.878 ± 0.190	2.062 (4)	7.82
	TX	3.554 (2.469–5.152) a	1.713 ± 0.199	4.076 (4)	6.62
Indoxacarb	Lab	0.124 (0.056–0.198) c	1.261 ± 0.225	3.354 (4)	—
	PH	1.411 (1.129–1.732) b	1.934 ± 0.185	1.936 (5)	11.38
	TX	1.977 (1.394–2.860) a	2.224 ± 0.239	5.545 (4)	15.94
Chlorantraniliprole	Lab	0.118 (0.031–0.226) c	1.145 ± 0.173	7.336 (5)	—
	PH	0.464 (0.347–0.592) b	1.556 ± 0.182	0.622 (4)	3.93
	TX	2.668 (2.188–3.300) a	1.739 ± 0.176	3.014 (4)	22.61
Cyantraniliprole	Lab	1.189 (0.465–1.991) ab	0.889 ± 0.192	3.227 (4)	—
	PH	2.571 (2.149–3.410) a	1.968 ± 0.198	4.756 (4)	2.16
	TX	1.018 (0.829–1.233) b	2.470 ± 0.272	0.860 (4)	0.86
Spinosad	Lab	0.555 (0.232–0.898) a	1.570 ± 0.231	5.136 (4)	—
	PH	0.669 (0.541–0.821) a	1.869 ± 0.188	3.578 (4)	1.21
	TX	0.693 (0.552–0.864) a	1.770 ± 0.181	2.835 (4)	1.25
Spinetoram	Lab	0.057 (0.015–0.106) c	1.220 ± 0.245	2.676 (4)	—
	PH	0.876 (0.686–1.082) a	1.853 ± 0.188	1.939 (5)	15.37
	TX	0.375 (0.175–0.557) b	2.158 ± 0.331	4.763 (4)	6.58
Emamectin benzonate	Lab	0.044 (0.027–0.061) c	1.440 ± 0.186	1.798 (5)	—
	PH	1.961 (1.639–2.335) a	2.528 ± 0.256	1.303 (4)	44.57
	TX	0.465 (0.370–0.569) b	1.962 ± 0.204	3.627 (4)	10.57
Chlorfluazuron	Lab	0.247 (0.132–0.384) b	0.816 ± 0.134	4.808 (5)	—
	PH	1.496 (1.180–1.863) a	1.703 ± 0.164	1.546 (5)	6.06
	TX	1.355 (1.074–1.651) a	2.176 ± 0.240	1.923 (4)	5.49
Lufenuron	Lab	0.383 (0.233–0.572) b	0.888 ± 0.139	4.156 (5)	—
	PH	1.722 (1.175–2.322) a	1.281 ± 0.175	2.055 (4)	4.50
	TX	0.511 (0.336–0.683) b	2.238 ± 0.268	4.333 (4)	1.33
Hexaflumuron	Lab	0.273 (0.163–0.401) b	0.962 ± 0.142	3.630 (5)	—
	PH	0.353 (0.271–0.441) b	1.920 ± 0.217	1.949 (4)	1.29
	TX	0.568 (0.415–0.757) a	2.053 ± 0.201	4.378 (4)	2.08
Methoxyfenozide	Lab	0.473 (0.271–0.742) b	1.382 ± 0.167	7.451 (5)	—
	PH	0.896 (0.591–1.242) a	1.798 ± 0.202	4.191 (4)	1.89
	TX	0.890 (0.651–1.153) a	1.427 ± 0.175	1.230 (4)	1.88

Different letters in each insecticide indicate significant differences among different populations.

**Table 4:** Virulence of *Spodoptera exigua* multiple nucleopolyhedrovirus (SeMNPV) against three populations of *S. exigua*.

Time	Population	LC_25_ (×10^6^ OBs/mL) (95% CI)	LC_50_ (×10^6^ OBs/mL) (95% CI)	Slope ± SE	χ^2^ (df)
48 h	Lab	1.790 (0.583–3.097)	4.602 (2.528–7.453)	1.645 ± 0.178	8.627 (4)
	PH	8.578 (6.570–10.642)	19.107 (15.645–23.676)	1.939 ± 0.196	2.944 (4)
	TX	-	-	-	-
72 h	Lab	1.563 (0.537–2.691)	4.314 (2.449–6.725)	1.530 ± 0.177	6.446 (4)
	PH	4.349 (2.189–6.603)	11.461 (7.729–16.755)	1.603 ± 0.175	5.023 (4)
	TX	23.643 (15.976–34.291)	138.900 (85.311–297.972)	0.877 ± 0.124	4.474 (5)
96 h	Lab	0.571 (0.022–1.502)	2.057 (0.410–4.008)	1.212 ± 0.175	8.547 (4)
	PH	0.535 (0.131–1.120)	1.929 (0.849–3.082)	1.211 ± 0.204	2.760 (4)
	TX	2.358 (1.053–3.960)	14.952 (10.293–21.208)	0.841 ± 0.111	3.804 (5)
120 h	Lab	0.182 (0.030–0.441)	0.717 (0.244–1.262)	1.135 ± 0.208	1.318 (4)
	PH	0.229 (0.022–0.660)	0.979 (0.229–1.942)	1.070 ± 0.216	1.217 (4)
	TX	1.966 (1.162–2.859)	6.312 (4.642–8.142)	1.331 ± 0.136	3.238 (5)

No larval mortality was observed in the population TX at 48 h.

**Table 5:** Resistance of *S. exigua* against eleven insecticides combining with SeMNPV.

Insecticide	Population	LC_50_ (mg/L) (95% CI)	Slope ± SE	χ^2^ (df)	RR ^a^	Fold ^b^
Chlorfenapyr + SeMNPV (LC_25_)	Lab	0.319 (0.254–0.392)	1.808 ± 0.171	4.046 (5)	0.59	0.59
	PH	0.319 (0.220–0.424)	1.391 ± 0.162	2.432 (5)	0.59	0.08
	TX	2.204 (1.889–2.575)	2.607 ± 0.216	2.396 (5)	4.10	0.62
Indoxacarb + SeMNPV (LC_25_)	Lab	0.239 (0.182–0.323)	1.345 ± 0.173	3.189 (4)	1.93	1.93
	PH	0.488 (0.358–0.659)	1.630 ± 0.153	5.319 (5)	3.94	0.35
	TX	0.577 (0.491–0.677)	2.240 ± 0.175	4.433 (5)	4.65	0.29
Chlorantraniliprole + SeMNPV (LC_25_)	Lab	0.334 (0.258–0.420)	1.591 ± 0.160	4.333 (5)	2.83	2.83
	PH	0.048 (0.027–0.071)	1.242 ± 0.170	4.273 (5)	0.41	0.10
	TX	1.065 (0.791–1.437)	1.818 ± 0.146	7.389 (5)	9.03	0.40
Cyantraniliprole + SeMNPV (LC_25_)	Lab	1.107 (0.839–1.520)	1.208 ± 0.135	1.971 (5)	0.93	0.93
	PH	0.381 (0.274–0.514)	1.742 ± 0.169	5.575 (5)	0.32	0.15
	TX	0.572 (0.435–0.734)	1.278 ± 0.147	1.904 (4)	0.48	0.56
Spinosad + SeMNPV (LC_25_)	Lab	0.699 (0.516–1.022)	1.183 ± 0.140	4.620 (5)	1.26	1.26
	PH	0.557 (0.432–0.740)	1.354 ± 0.141	2.798 (5)	1.00	0.83
	TX	0.465 (0.358–0.599)	1.180 ± 0.120	4.339 (5)	0.84	0.67
Spinetoram + SeMNPV (LC_25_)	Lab	0.035 (0.019–0.051)	1.277 ± 0.177	3.374 (5)	0.61	0.61
	PH	0.270 (0.215–0.332)	1.826 ± 0.178	1.841 (5)	4.74	0.31
	TX	0.142 (0.109–0.175)	1.890 ± 0.200	1.327 (5)	2.49	0.38
Emamectin benzonate + SeMNPV (LC_25_)	Lab	0.094 (0.044–0.174)	0.525 ± 0.117	0.905 (5)	2.14	2.14
	PH	0.125 (0.086–0.166)	1.562 ± 0.187	0.976 (5)	2.84	0.06
	TX	0.379 (0.306–0.458)	1.936 ± 0.184	0.669 (5)	8.61	0.81
Chlorfluazuron + SeMNPV (LC_25_)	Lab	0.049 (0.021–0.082)	0.797 ± 0.131	2.381 (5)	0.20	0.20
	PH	0.237 (0.177–0.303)	1.602 ± 0.169	3.559 (5)	0.96	0.16
	TX	1.080 (0.715–1.625)	2.168 ± 0.234	7.577 (4)	4.37	0.80
Lufenuron + SeMNPV (LC_25_)	Lab	0.361 (0.218–0.634)	1.102 ± 0.137	7.050 (5)	0.94	0.94
	PH	0.367 (0.240–0.506)	1.221 ± 0.149	2.862 (5)	0.96	0.21
	TX	1.099 (0.856–1.436)	1.391 ± 0.146	3.014 (5)	2.87	2.16
Hexaflumuron + SeMNPV (LC_25_)	Lab	0.145 (0.088–0.217)	0.823 ± 0.127	2.456 (5)	0.53	0.53
	PH	0.130 (0.087–0.176)	1.508 ± 0.186	1.393 (5)	0.48	0.37
	TX	0.415 (0.297–0.570)	1.880 ± 0.192	4.703 (4)	1.52	0.73
Methoxyfenozide + SeMNPV (LC_25_)	Lab	0.171 (0.138–0.209)	2.071 ± 0.206	2.946 (5)	0.36	0.36
	PH	0.141 (0.085–0.203)	1.159 ± 0.153	4.133 (5)	0.30	0.16
	TX	0.739 (0.617–0.883)	2.376 ± 0.217	2.938 (5)	1.56	0.83

^a^ RR = resistance ratio (LC_50_ of the insecticide against three populations with SeMNPV infection/LC_50_ of the insecticide against the Lab population without SeMNPV infection), ^b^ Fold = RR of the insecticide combining with SeMNPV/RR of the insecticide.

## Data Availability

The data presented in this study are available on request from the corresponding author.
